# Supplementary material for: Gαi2 Signaling Regulates Neonatal Respiratory Adaptation
Source: Int J Mol Sci. 2025 Nov 1;26(21):10655. doi: 10.3390/ijms262110655 (PMC12610543; doi:10.3390/ijms262110655)
Supplement: Supplementary file 1 [file ijms-26-10655-s001.zip › ijms-3831308-supplementary.pdf]

Supplement

Suppl. Table S1. Average litter size of *Gnai2*-matings.

Mating strategy	∅ litter
$Gnai2^{+/+}$ x $Gnai2^{+/+}$	6.3 ± 2.5 (n=11)
$Gnai2^{+/-}$ x $Gnai2^{+/-}$	5.6 ± 3.2 (n=47)
$Gnai2^{-/-}$ x $Gnai2^{-/-}$	3.5 ± 2.2 (n=13)

Suppl. Table S2. Mendelian ratio of littermates born from heterozygous *Gnai2*-matings on a 129/Sv background. Statistics is based on Chi-square analyses.

Genotype	P21	% expected
$Gnai2^{+/+}$	177 (29.9%)	25
$Gnai2^{+/-}$	356 (60.1%)	50
$Gnai2^{-/-}$	59 (10.0%)	25
	592 (116 litter)	
p value	n.s.	

Suppl. Table S3. Mendelian ratio of P21 littermates born from heterozygous *Gnai3*-matings on a C57BL/6N or 129/Sv background. Statistics is based on Chi-square analyses.

Genotype	C57BL/6N	129/Sv	% expected
$Gnai3^{+/+}$	38 (24.5%)	29 (19.1%)	25
$Gnai3^{+/-}$	78 (50.3%)	85 (55.9%)	50
$Gnai3^{-/-}$	39 (25.1%)	39 (25%)	25
	155 (18 litter)	152 (31 litter)	
p value	n.s.	n.s.	

Suppl. Table S4. Average litter size of *Gnai3*-matings.

Mating strategy	∅ litter
$Gnai3^{+/+}$ x $Gnai3^{+/+}$	5.1 ± 1.5 (n=8)
$Gnai3^{+/-}$ x $Gnai3^{+/-}$	6.4 ± 2.1 (n=22)
$Gnai3^{-/-}$ x $Gnai3^{-/-}$	4.9 ± 3 (n=8)

Suppl. Table S5. Mendelian ratio of littermates born from *Gnai2*$^{fl/fl}$; *Gnai3*$^{fl/+}$; *ShhCre*$^{+/+}$ X *Gnai2*$^{fl/fl}$ *Gnai3*$^{fl/fl}$; *ShhCre*$^{tg/+}$-matings. Statistics is based on Chi-square analyses.

**Genotype**	**P21**	**% expected**
Gnai2$^{fl/fl}$; *Gnai3*$^{fl/+}$	14 (17.5%)	25
Gnai2$^{fl/fl}$; *Gnai3*$^{fl/fl}$	25 (31.25%)	25
Gnai2$^{fl/fl}$; *Gnai3*$^{fl/+}$; *ShhCre*$^{tg/+}$	24 (30%)	25
Gnai2$^{fl/fl}$; *Gnai3*$^{fl/fl}$; *ShhCre*$^{tg/+}$	15 (18,75%)	25
	80 (8 litter)	
p value	n.s.	

Suppl. Table S6. Area under the curve of the time-response curves of the depicted parameters of wt and *Gnai2*$_{(s)}^{-/-}$ mice shown in Figure 8.

**AUC (0 - 120 min)**	**wt**	***Gnai2***$_{(s)}^{-/-}$	**p-value**
resistance [(cmH$_2$O/ml/sec) min]	86.2±2.3	83.5±1.5	n.s.
pulmonary artery pressure [cmH$_2$O min]	262.3±34.3	379.6±79.6	n.s.
max. insp. flow [(µl/sec) min]	356600±18120	414200±4810	$p < 0.05$
max. exp. flow [(µl/sec) min]	274400±11910	326500±8837	$p < 0.01$
tidal volume [ml min]	20050±1380	27050±1398	$p < 0.01$
compliance [(µl/cmH$_2$O) min]	3633 ± 260.9	4852 ± 258.3	$p < 0.01$

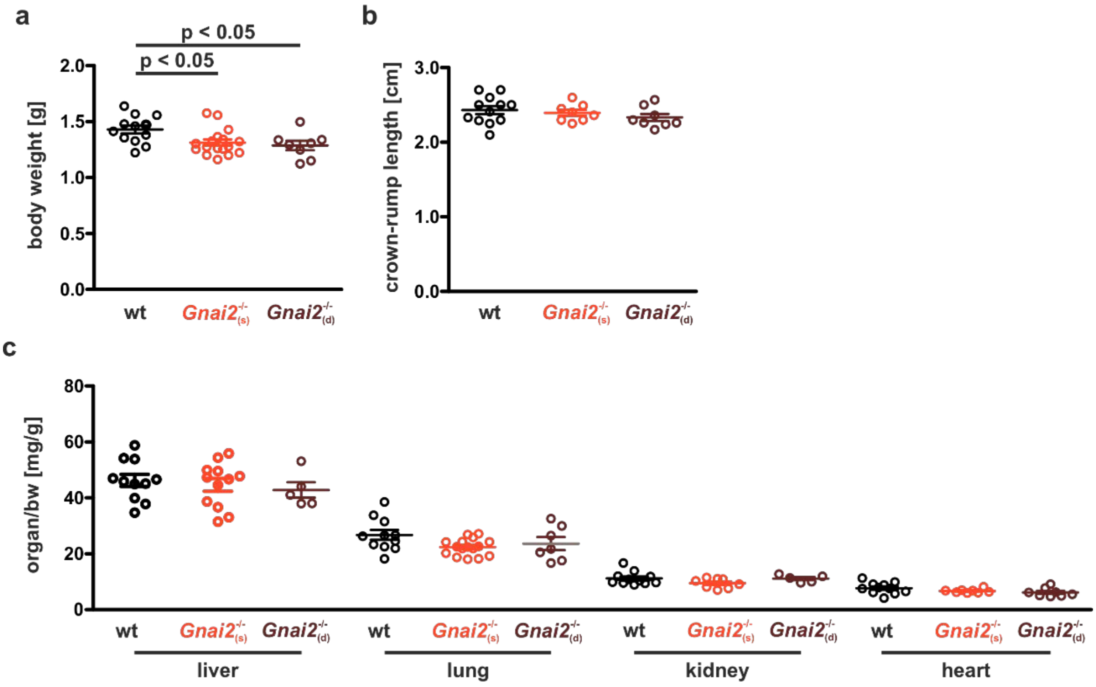

Supplementary Figure S1. Somatometric and organometric analysis of neonatal mice. (**a**) Body weight of new-born wt (black), Gnai2$^{-/-}$$_{(s)}$ (red) and Gnai2$^{-/-}$$_{(d)}$ (brown) mice was significantly reduced compared to wt animals. (**b**) Crown-rump length and (**c**) organ to body weight ratio of of new-born wt, Gnai2$^{-/-}$$_{(s)}$ and Gnai2$^{-/-}$$_{(d)}$ mice were equal. The data shown were obtained from 5 to 16 animals per group. Mean ± SEM. # = $p < 0.05$ Student's t test between Gnai2$^{-/-}$$_{(s)}$ and Gnai2$^{-/-}$$_{(d)}$.

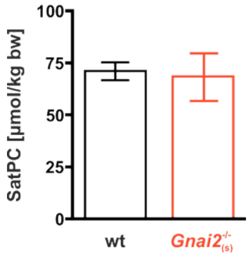

Supplementary Figure S2. Quantification of saturated phosphatidylcholine (SatPC) in lung tissue. SatPC levels of new-born wt and *Gnai2*$^{-/-}$ mice were equal. Mean ± SEM.

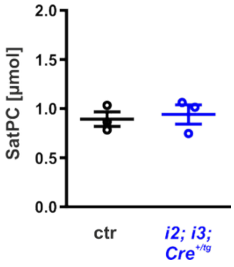

Supplementary Figure S3. Quantification of saturated phosphatidylcholine (SatPC) in lung tissue of Alveolar Type II cell-specific *Gnai2; Gnai3* double-deficient mice. SatPC levels of 11-week-old control (*Gnai2*$^{fl/fl}$; *Gnai3*$^{fl//fl}$) and double-deficient *Gnai2*$^{fl/fl}$; *Gnai3*$^{fl/fl}$; *ShhCre*$^{+/tg}$ mice. Mean ± SEM.
